# Crystallographic Analysis of Ground and Space Thermostable T1 Lipase Crystal Obtained via Counter Diffusion Method Approach

**DOI:** 10.1155/2014/904381

**Published:** 2014-01-02

**Authors:** Sayangku Nor Ariati Mohamad Aris, Adam Leow Thean Chor, Mohd Shukuri Mohamad Ali, Mahiran Basri, Abu Bakar Salleh, Raja Noor Zaliha Raja Abd. Rahman

**Affiliations:** ^1^Enzyme and Microbial Technology Research Centre, Faculty of Biotechnology and Biomolecular Sciences, Universiti Putra Malaysia, 43400 Serdang, Selangor, Malaysia; ^2^Department of Microbiology, Faculty of Biotechnology and Biomolecular Sciences, Universiti Putra Malaysia, 43400 Serdang, Selangor, Malaysia; ^3^Department of Cell and Molecular Biology, Faculty of Biotechnology and Biomolecular Sciences, Universiti Putra Malaysia, 43400 Serdang, Selangor, Malaysia; ^4^Department of Biochemistry, Faculty of Biotechnology and Biomolecular Sciences, Universiti Putra Malaysia, 43400 Serdang, Selangor, Malaysia; ^5^Department of Chemistry, Faculty of Science, Universiti Putra Malaysia, 43400 Serdang, Selangor, Malaysia

## Abstract

Three-dimensional structure of thermostable lipase is much sought after nowadays as it is important for industrial application mainly found in the food, detergent, and pharmaceutical sectors. Crystallization utilizing the counter diffusion method in space was performed with the aim to obtain high resolution diffracting crystals with better internal order to improve the accuracy of the structure. Thermostable T1 lipase enzyme has been crystallized in laboratory on earth and also under microgravity condition aboard Progress spacecraft to the ISS in collaboration with JAXA (Japanese Aerospace Exploration Agency). This study is conducted with the aims of improving crystal packing and structure resolution. The diffraction data set for ground grown crystal was collected to 1.3 Å resolution and belonged to monoclinic *C2* space group with unit cell parameters *a* = 117.40 Å, *b* = 80.95 Å, and *c* = 99.81 Å, whereas the diffraction data set for space grown crystal was collected to 1.1 Å resolution and belonged to monoclinic *C2* space group with unit cell parameters *a* = 117.31 Å, *b* = 80.85 Å, and *c* = 99.81 Å. The major difference between the two crystal growth systems is the lack of convection and sedimentation in microgravity environment resulted in the growth of much higher quality crystals of T1 lipase.

## 1. Introduction

Lipases (triacylglycerol acylhydrolase) are enzymes which are present in many different organisms. It catalyze both the hydrolysis of triglycerides and the synthesis of esters formed from alcohol and long chain fatty acids. Enzymes from thermophiles often show more stability towards organic solvents and exhibit higher activity at elevated temperatures. Thus, this thermostable enzyme became an important requirement as a biocatalyst in industry including detergent, food, pharmaceuticals, cosmetic, textiles, fine chemicals, and biodiesel [1]. To better understand the mechanism of the enzyme, it is important to elucidate the three-dimensional structure of the enzyme. Performing precise X-ray data collection from high quality single crystal is the suitable way to solve the 3D structure accurately. The bottleneck of X-ray crystallography is obtaining high quality crystal which is influenced by the protein's purity and how the crystals are grown [2]. An excellent crystallization method called the counter diffusion method was introduced by García-Ruiz and Moreno [3]. This technique requires a convection-free environment, which can be achieved using either gelled solutions, very thin capillaries, or microgravity conditions. The time for crystal growth may be extended by using longer gel tube. Furthermore, it is also possible to obtain larger crystals suitable for X-ray diffraction if capillaries with larger diameter are used [4]. There are two main benefits of growing crystals in counter diffusion method rather than vapour diffusion. The first benefit is the convection disturbances such as the effect of solution density or the fluctuating temperature which can be better controlled. The second benefit is the absence of crystal sedimentations. Sedimentation effect will result in the formation of crystals which usually grew at the bottom of the growth cell. By performing the crystallization inside a capillary, it can minimize or even suppress convection in solution on earth and create diffusive environment where capillary forces counterbalance gravity forces [5]. One of the challenges to get higher quality crystals is the natural convection that exists under normal earth gravity. On earth, gravity often has a negative impact on growing protein crystals. In microgravity, however, gravitational disturbances are at minimum level, thus allowing some crystals to grow in a more regular and perfect manner. Approach of crystallization in space using counter diffusion method has been conducted in order to reduce buoyancy-driven convection and avoid sedimentation of crystals. It will improve the yield of high quality crystals with better internal order which is suitable for diffraction analysis and thus give more accurate and reliable protein structure. An important objective for determining the three-dimensional structure of protein is to understand the structural mechanism and their biological activity. The ultimate goal of this study is to improve the crystal quality which will result in more accurate three-dimensional protein structures. Thus it will lead to a more precise understanding of biological function.

## 2. Materials and Methods

### 2.1. Materials

Resins for purification were purchased from GE Healthcare (Sweden). Capillary C-Tube Crystal-Tube Kit was purchased from Confocal Science Inc.

### 2.2. Purification of T1 Lipase

*Geobacillus zalihae* strain T1 lipase was overexpressed in pGEX vector in the prokaryotic system. Purification of T1 lipase was conducted according to the procedures of Leow et al. [6]. Mature T1 lipase was then purified via additional step, anion exchange chromatography using Q-sepharose HP column. The protein was eluted with 25 mM Tris-HCl (pH 9) containing linear gradient of 0 to 0.1 M sodium chloride at a flow rate of 0.5 ml/min. This step was applied to remove the remaining trace of impurities from earlier step. The purity of the fractions with high lipase activity was analysed by SDS-PAGE [7] and Native-PAGE. The desired protein was then desalted and kept in the storage buffer (25 mM Tris-HCl, pH 8).

### 2.3. Crystallization of T1 Lipase

Previously, crystallization of T1 lipase was achieved by sitting drop vapour diffusion method using reservoir solution composed of 1 M NaCl, 0.1 M NaH_2_PO_4_, 0.1 M KH_2_PO_4_, and 0.1 M MES pH 6.5 [8]. In the experiment described here, gel-tube method [4] which was modified from the original capillary counter diffusion method of García-Ruiz and Moreno [3] was applied for crystallization in order to control and improve the crystal quality. The glass capillaries of 0.5 mm in diameter and 60 mm in length from Crystal-Tube Kit (Confocal Science Inc.) were loaded with approximately 8 *μ*L mixture of protein solution (5 mg/mL) and precipitant of 1 : 1 ratio. The upper end of the capillary was sealed with clay. The other end was inserted into the presoaked gel. The gel tube was cut to a length of 8 mm after being equilibrated by soaking it into the formulation of reservoir. The capillary was then placed in the cylinders of the syringe cases and the bottom of the cylinder cases was covered with its caps using a forceps. The caps of the cylinders of the syringe cases were carefully sealed. The area around the capillaries that is attached to the cylinder cases and the upper end of the capillaries were sealed with Araldite adhesive (Huntsman Advance Material). The crystallization of T1 lipase on the ISS was carried out in the Japanese Experiment Module Kibo of the JAXA (Japanese Aerospace Exploration Agency). The space sample unit aboard the Progress spacecraft to ISS. The canister containing the space unit samples was mounted to the experiment facility, the PCRF (Protein Crystallization Research Facilities) by a crew member. The temperature for both space and ground control was set to 20°C.

### 2.4. X-Ray Diffraction Analysis

Crystals of T1 lipase were mounted in cryoloop soaked in cryoprotectant solution consisting of 40% glycerol and then flash-cooled in a liquid nitrogen stream at 100 K. High resolution X-ray diffraction data for both space grown crystal and ground control of T1 lipase crystals were collected using Rayonix MAR225HE CCD detector at station BL41XU of SPring-8 (Harima, Japan). The detector was set at a distance of 130 mm with 0.5 seconds per frame exposure and a 0.5° per frame oscillation was used. All resulting data from this experiment were integrated and scaled using the programs DENZO and SCALEPACK from the HKL-2000 suite [9].

### 2.5. Structure Determination

Structure refinement and model building were performed by using CCP4 program suite (Collaborative Computational Project, Number 4 and COOT (Crystallographic Object-Oriented Toolkit). Meanwhile, structural analysis was carried out using the YASARA (Yet Another Scientific Artificial Reality Application) software version 10.2.1. [10].

## 3. Results and Discussion

**Figure 1:**
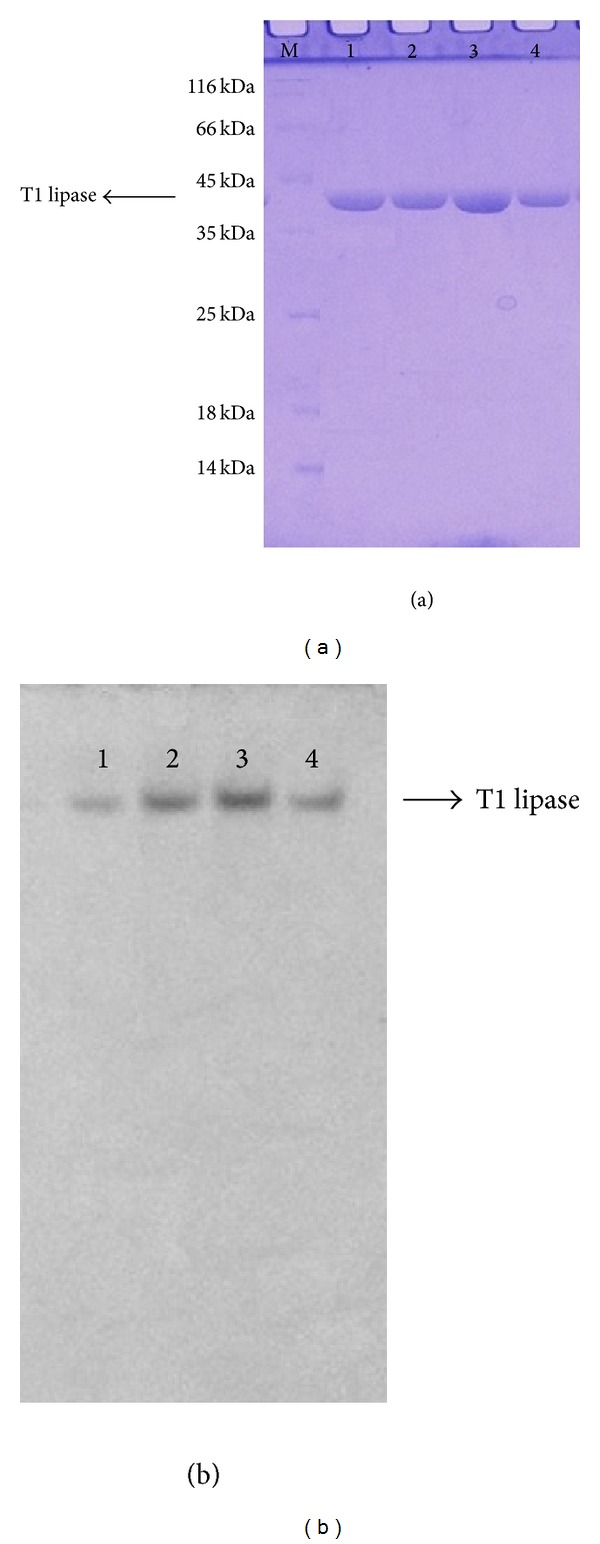
Detection of T1 lipase on gel electrophoresis. (a) SDS-PAGE of T1 lipase after ion exchange chromatography (lane: M: marker, 1–4: purified T1 lipase). (b) The homogeneity was confirmed via Native-PAGE. (lane: 1–4: purified T1 lipase).

**Figure 2:**
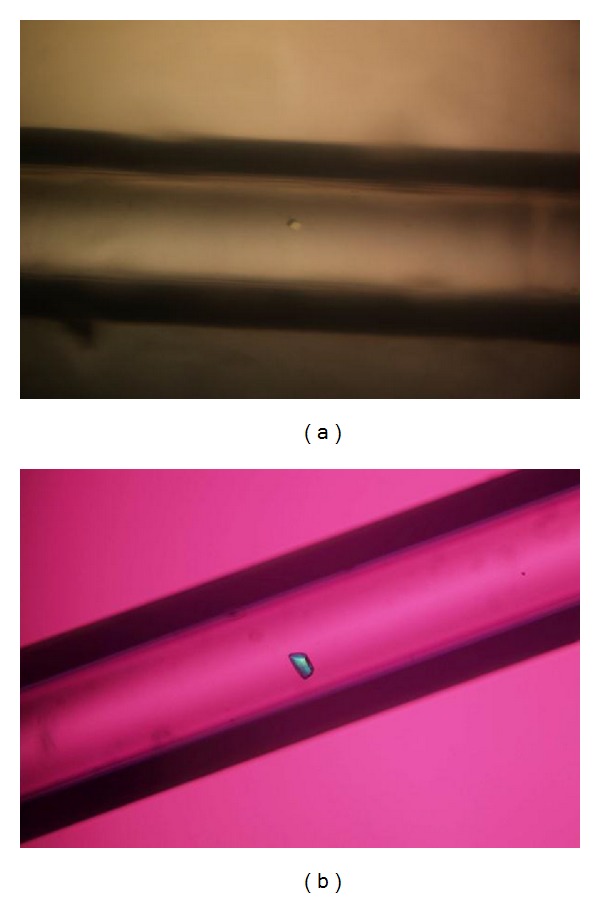
Ground-grown (a) and space-grown (b) crystals of T1 lipase in capillary using counter diffusion method.

**Figure 3:**
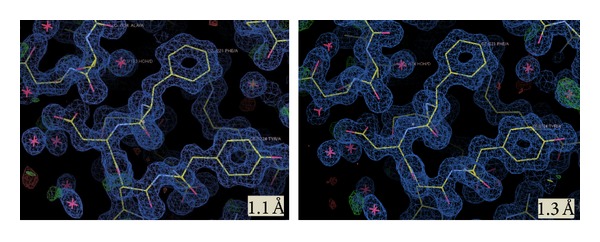
Snapshots of ground-grown crystal (1.1 Å) and space-grown crystal (1.3 Å) electron density map countered at 1 sigma level displayed in COOT.

**Figure 4:**
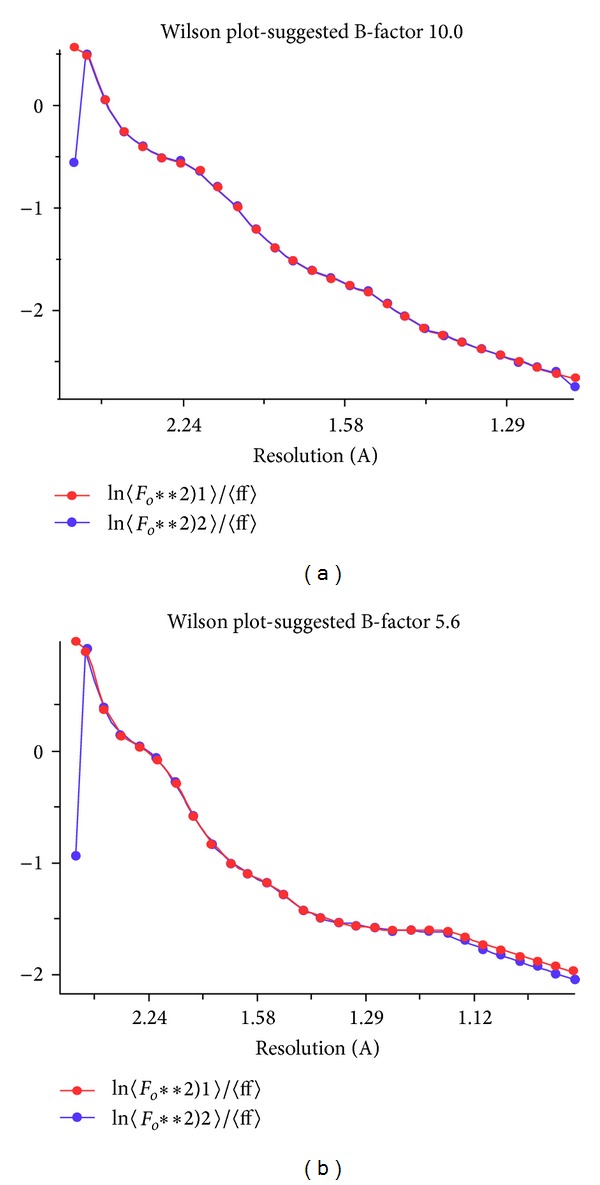
Wilson plot for (a) ground-grown structure and (b) space-grown structure.

**Figure 5:**
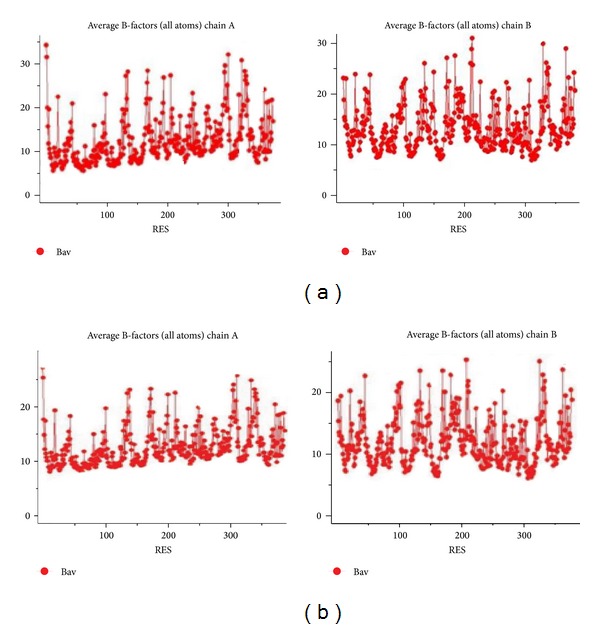
Plot of the average B-factor per residue for main chain of each structure. (a) Ground-grown crystal structure and (b) space-grown crystal structure.

**Figure 6:**
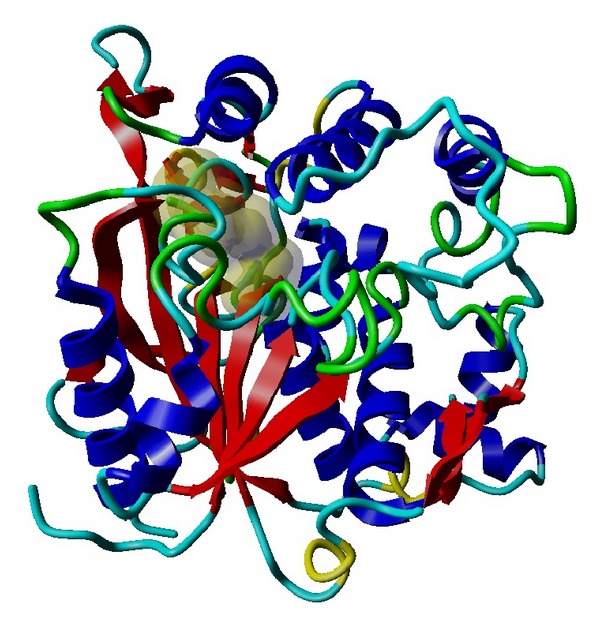
Ribbon diagram for overall structure. The structure of T1 lipase is shown as a ribbon diagram with *α*-helices (blue), *β*-strands (red), 3^10^ helix (yellow), turn (green), and coil (cyan). It comprises the catalytic triad Ser113, Asp317, and His358. (Comparison of molecules A and B for both structures shows that the overall structures of these two molecules are almost identical. Therefore, only one of them is shown.)

**Figure 7:**
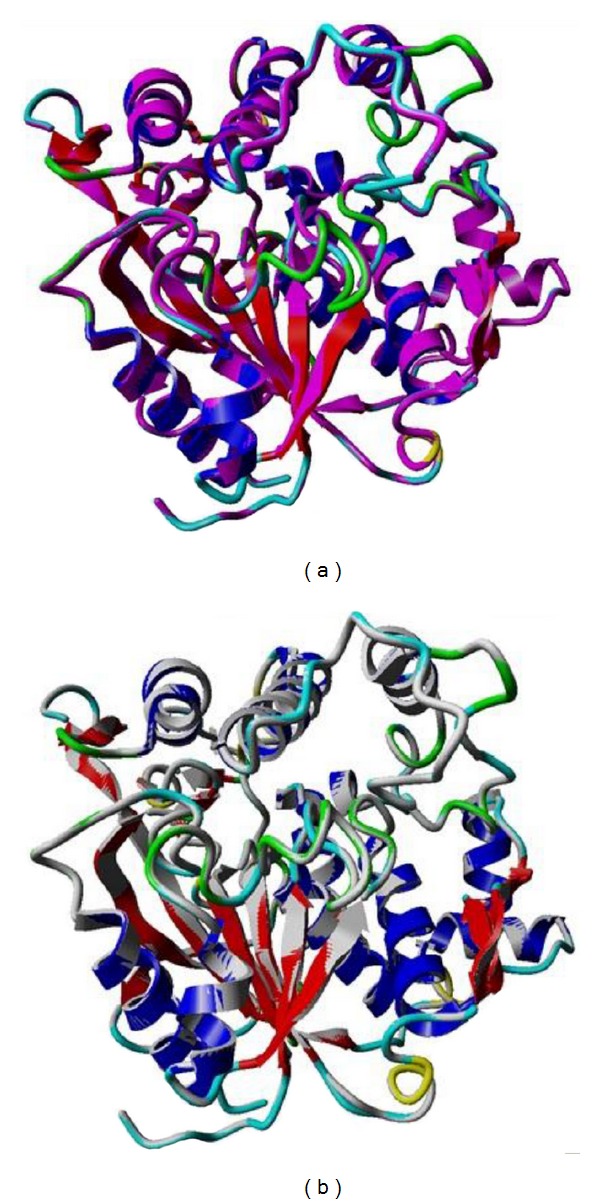
Superimpose of chains A and B for ground-grown and space-grown of T1 lipase. (a) Superimpose of chain A for both T1 lipase crystal (magenta for space crystal) with RMSD of 0.2185 Å. (b) Superimpose of chain B (gray for space crystal) with RMSD of 0.4214 Å.

T1 lipase was successfully purified to homogeneity with a molecular weight of 43 kDa. The purity was confirmed by the presence of a single band on SDS-PAGE and Native-PAGE (Figure 1). T1 lipase was extensively purified via ion exchange chromatography to gain higher purity and to improve its uniformity. The purity and uniformity of the protein sample are paramount important in crystallization. Incorporation of the impurities or nonuniformity in the crystal will cause lattice strain and reduce the crystalline order, which will eventually decrease the quality of the diffraction pattern and thus become unsuitable for X-ray structural analysis [11]. To date, very few thermostable lipase crystals had been reported. Crystals from thermostable *Bacillus stearothermophilus* P1 lipase [12], *Bacillus thermocatenulatus* lipase 2 (BTL2) [13], and *Bacillus stearothermophilus* L1 (L1 lipase) [14] were obtained from hanging drop vapour diffusion method. Currently, only two thermostable lipases were reported grown by counter diffusion method, which are L42 lipase and L2 lipase. X-ray data of L42 lipase were collected at 2.0 Å and the crystal belonged to the monoclinic space group [15]. However thermostable L2 lipase crystal belonged to orthorhombic space group with resolution 2.7 Å [16]. Maruki et al. [17] have acquired a high resolution structure of a new crystal form of human triosephosphate isomerase (TIM) by employing counter diffusion method crystallization in microgravity environment.  Figure 2 shows T1 lipase crystal obtained by counter diffusion method (ground and space grown). The dimensions of the crystals were approximately 0.08 × 0.04 × 0.04 mm and 0.16 × 0.08 × 0.04 mm for ground-grown crystal and space-grown crystal, respectively. Data sets for T1 lipase crystals were collected at the beamline of the BLX41 at SPring-8 Synchrotron radiation facilities, Japan, to a resolution of 1.3 Å for ground-grown crystal and 1.1 Å for space-grown crystal.  Table 1 shows X-ray data statistics for T1 lipase crystals grown under microgravity and on the earth. The reflections of ground-grown crystal was indexed on a centred monoclinic lattice (*C2* with unit cell parameters *a* = 117.40 Å, *b* = 80.95 Å, *c* = 99.81 Å, *α* = 90°, *β* = 96.76°, and *γ* = 90°). Space-grown crystal belonged to the same system with unit cell parameters *a* = 117.31 Å, *b* = 80.85 Å, *c* = 99.81 Å, *α* = 90°, *β* = 96.87°, and *γ* = 90°. Mosaicity measurements and the resolution of the diffraction are the most important indicators for crystal quality [18]. Space-grown crystals display a better signal to noise ratio (I/Sigma) rather than ground-grown crystals. It shows that microgravity can provide a better environment for growing good quality crystals which are difficult to obtain on the ground. The quantity and quality of the diffraction data will affect the electron density map. The difference in resolution will be reflected in the quality of the electron density map, where the model of three-dimensional protein structures is built. A higher resolution of the data results in higher resolution of the electron density map. It will give higher accuracy of the positions of the atoms in the structure. From the experiment, the final 2Fo-Fc electron density maps which are of excellent quality in both crystals are further improved in space-grown crystals. This is due to the higher resolution of space-grown crystal. The positions of the atoms are much better defined. Well-defined side chains of amino acids and the greater number of ordered water molecules are emphasized, thus resulting in more accurate structure [19]. Overall, the polypeptide backbone can be easily fit into the electron density observed with space data.  Figure 3 shows the snapshots of electron density from space-grown and ground-grown crystal obtained after final refinement. All atoms in the protein model were best fit into the electron density map. The crystal has a predicted solvent content of 55.11% and a Matthews's coefficient value (*V*_*m*_) of 2.74 Å^3^Da^−1^ for ground grown crystal and solvent content of 55.01% and a Matthews coefficient value (*V*_*m*_) of 2.73 Å^3^Da^−1^ for space grown crystal. The *V*_*m*_ and solvent content values of T1 lipase are in the range, as Matthews observed that the solvent content in protein crystal ranged from 27% to 65% with an average of 43% [20]. According to Kantardjieff and Rupp, protein crystals with less solvent tend to diffract better [21]. It shows that tightly packed crystals tend to diffract better than the loosely packed ones. In early stages of crystal structure determination, it is important to know the solvent content which helps to determine the number of molecules in the asymmetric unit [20]. The atomic coordinates *Geobacillus zalihae* T1 lipase (Protein Data Bank under accession code 2DSN) without water molecules was used as a template for the refinement of the ground-grown and space-grown crystal structures. The structural refinement was performed using the molecular replacement method using CCP4 program suite (Collaborative Computational Project, number 4). Refinement was performed by alternating rounds of model building using the program COOT (Crystallographic Object-Oriented Toolkit) and crystallographic refinement using REFMAC5. The quality and accuracy of the refined model was evaluated using Ramachandran plot [22] and ERRAT. Analysis of B-factor will provide information of the protein dynamics, flexibility of amino acids, and protein stability [23]. Crystal packing interactions make a significant contribution to the value of B-factor [24]. The B-factor (atomic displacement parameter) in protein crystal structures reflects the fluctuation of an atom about its average position. The distribution of B-factors along a protein sequence is regarded as an important indicator of the protein's structure, reflecting its flexibility and dynamicity, where the low value of B-factors correspond to well-defined parts of the structure, whereas high value of B-factors might indicate highly disordered parts of the structure or even misinterpreted parts of the model [25].  Figure 4 shows that Wilson plot generated from CCp4 software gives overall value B-factor of 10 A^2^ and 5.6 A^2^ for ground-grown and space-grown crystal structure, respectively. Overall, ground-grown crystal structure shows higher B-factor value, indicating that it has the higher flexibility compared to the space-grown structures. The distribution of average B-factors over the peptide main-chain for each molecule for both structures are shown in Figure 5.  Table 2 shows refinement statistics for ground-grown and space-grown crystals. Both refined model contains two molecules (chain A and chain B) per asymmetric unit and each comprises 387 amino acids (residues Ser2 to Pro388) (Figure 3). Both solved structures show a common *α*/*β* hydrolase fold that comprises the conserved Ser-His-Asp catalytic triad (Ser113-His358-Asp317) (Figure 6). The arrangement of *α*-helix 6 and *α*-helix 7 was shown as “lid” that covers the active site. The presence of hydrophobic region of *α*-helix 6 (Phe176, Phe180, Phe181, Leu183, Ala186, Val187, Leu188, and Ala190) provided a hydrophobic surroundings to the active site. This environment may play an important role in enzyme catalysis. The two molecules in the asymmetric unit are highly similar to each other. Main backbone of chain A and chain B for both ground- and space-grown crystal was superimposed giving root mean square deviation (RMSD) value of 0.2185 Å for chain A and 0.4214 Å for chain B, respectively (Figure 7).

## 4. Conclusion

Comparative crystallographic analysis revealed that enhanced quality of the diffraction data was derived from space-grown crystal. Compared to ground-grown crystal, the space-grown crystal diffracts better. As a result, an improved initial electron density map was obtained for modeling and refinement, contributing to clearer structural information of the enzyme. Strictly comparative crystallographic analysis reveals that space-grown crystals are better compared to ground-crystal. Analysis of data collection and refinement statistics showed that crystallization in counter diffusion method using microgravity environment have highly improved the internal order of crystals and thus gave a more precise three-dimensional structure. A more in depth analysis of both structures can help in the understanding of the enzyme.

## Figures and Tables

**Table 1:** X-ray data collection statistics for T1 lipase.

	Ground	Space
Space group	C2	C2
Unit cell parameters (Å)	*a* = 117.40	*a* = 117.31
	*b* = 80.95	*b* = 80.85
	*c* = 99.81	*c* = 99.81
Resolution range (Å)	30.0–1.30	40–1.10
*R*_merge_	0.103 (0.00)	0.063 (0.46)
Data completeness (%)	99.6 (99.1)	98.1 (94.1)
*I*/Sigma (I)	35.02 (3.93)	24.23 (2.0)
Mosaicity range	0.12–0.21	0.14–0.20
Molecule per asymmetric unit	2	2
Matthews coefficient (Å^3^/Da)	2.74	2.73
Solvent content (%)	55.11	55.01

Values in bracket refer to the highest resolution shell.

**Table 2:** Final statistics of refined structure for the ground-grown and space-grown T1 lipase crystals.

	Ground-grown crystal	Space-grown crystal
Resolution range used in refinement (Å)	30–1.2	30–1.2
Number of reflections used in refinement	272755	271607
*R* factor	0.134	0.129
*R* free	0.162	0.150
Number of protein atoms	387	387
Number of water molecules	721	781
Number of metal ions	7	7
Number of glycerols	4	4
*RMS deviation *		
Bond length (Å)	0.021	0.020
Angles (Deg)	1.882	1.822
Planes (Å)	0.014	0.014
*Ramachandran plot details *		
Residues in favoured regions (%)	91.5	91.8
Residues in additional allowed regions (%)	7.9	7.6
Residues in generously allowed regions (%)	0.3	0.3
Residues in disallowed regions (%)	0.3	0.3
*Average B-factor value (A* ^ 2^)		
Chain A	10.8	9.4
Chain B	12.1	10.5
